# Marine Microbial Metagenomics: From Individual to the Environment

**DOI:** 10.3390/ijms15058878

**Published:** 2014-05-19

**Authors:** Ching-Hung Tseng, Sen-Lin Tang

**Affiliations:** 1 Bioinformatics Program, Taiwan International Graduate Program, Institute of Information Science, Academia Sinica, Taipei 11529, Taiwan; E-Mail: chtzeng@gmail.com; 2 Biodiversity Research Center, Academia Sinica, Taipei 11529, Taiwan; 3 Institute of Biomedical Informatics, National Yang-Ming University, Taipei 11221, Taiwan

**Keywords:** bacteria, environmental genomics, metagenomics, microbial ecology

## Abstract

Microbes are the most abundant biological entities on earth, therefore, studying them is important for understanding their roles in global ecology. The science of metagenomics is a relatively young field of research that has enjoyed significant effort since its inception in 1998. Studies using next-generation sequencing techniques on single genomes and collections of genomes have not only led to novel insights into microbial genomics, but also revealed a close association between environmental niches and genome evolution. Herein, we review studies investigating microbial genomics (largely in the marine ecosystem) at the individual and community levels to summarize our current understanding of microbial ecology in the environment.

## 1. Introduction

The term “metagenome” was first coined by Jo Handelsman and her colleagues to cover a collection of environmental DNA from microbes that cannot be cultured in the laboratory [1]. These organisms represent the vast unseen majority of microbes on earth [2]. Thus, “metagenomics” refers to the study of metagenomes, also known as environmental genomics, ecological genomics, or community genomics. Currently, two major types of metagenomics studies prevail in public literature databases in terms of research targets, one being the community survey that resolves community composition or dynamics using phylogenetic marker genes like the 16S ribosomal RNA (rRNA) sequence, and the other being the functional metagenomics survey that studies the metabolic potential embedded in coding genes obtained from an environment. Both types of studies usually relate ecological associations to environmental conditions.

The history of studying environmental microbes using culture-independent methods can be traced back to the application of the fosmid library, developed by DeLong’s group, to clone large genomic DNA fragments into a fosmid vector maintained in an *Escherichia coli* surrogate host [3]. Screening, sequencing, and analyzing those partial microbial genomes extend our understanding into previously untapped microbiological territory. The next boost to metagenomics research was the use of the whole genome shotgun (WGS) approach employing next generation sequencing to bypass the need for cloning and direct sequencing of metagenomic DNA fragments in a high-throughput manner [4]. The massive decrease in sequencing costs in recent years further promises to dramatically increase access to genomic and metagenomic data.

**Figure 1.**
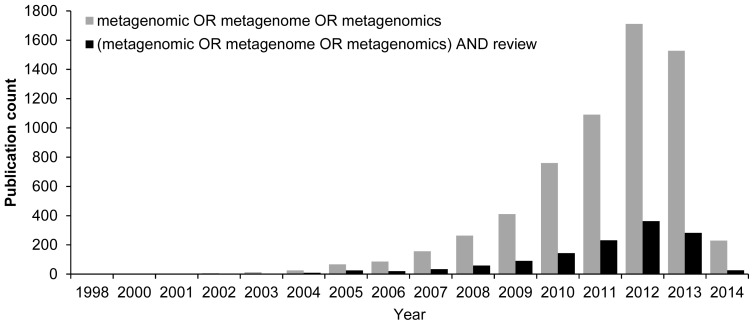
Number of publications related to metagenomics in the PubMed database from 1998. Different colors represent results using different search terms, which are labeled as color keys.

Since 1998, attention to metagenomics has gradually increased around the world, as witnessed by the growing number of related reports and even reviews (Figure 1) in the PubMed database [5]. Being a young and vibrant field of research, the immense scope of metagenomics cannot be fully covered in one paper, therefore we selected studies considered representative to each section discussed below, and apologize to the authors of studies that are relevant but not included herein.

Metagenomics has come a long way since its early days in the 21st century. Several comprehensive review articles have provided valuable summaries and perspectives at each stage in the development of metagenomics [6–14]. Although metagenomes in engineered and human-associated environments have been well-investigated, this overview deals mainly with marine microbes from individual genomics to community metagenomics. For individual genomics, we firstly summarize milestone discoveries derived from marine cyanobacteria, one of the important players in global carbon cycling, and list key findings in the surface-ocean and deep-sea microbes. For community metagenomics, we begin with reviewing studies probing plasmid metagenomes, which have received more attention in recent years, and then we present the ecological importance of environmental microbes by summarizing their special metabolisms for element cycling and genomic insights into communities inhabiting various oceanic regions. Finally, we discuss the decoupling of microbial community compositions and their functional potentials, which seems prevalent in the environment.

## 2. Environmental Ecology Derived from Functional Genomics

Cyanobacteria are important carbon and nitrogen fixers in our environment, and thus are of ecological significance in global geochemical cycling. Their remarkable abundance in the ocean further makes cyanobacteria excellent models for studying interactions between bacterial hosts and phages, which in turn can affect the genetic and functional diversity of the ocean gene pool. One exciting discovery of the past decade using genomics technology is the detection of the photosynthesis *psbA* gene in a cyanophage (*i.e.*, a virus capable of infecting cyanobacteria) genome. The phage-encoded *psbA* gene is transcribed during infection, presumably to bolster host photosynthesis by replacing the non-functional *psbA* gene in hosts and maintain energy generation until phage replication is complete [15]. Additional studies on two widespread marine cyanobacteria, *Synechococcus* and *Prochlorococcus*, and their cyanophages showed that photosynthesis genes commonly exist in cyanophage genomes, and significant genetic exchanges occur from host to phage, phage to host, and within the phage gene pool. Interestingly, the genetic exchanges involving cyanophages could have influenced the make-up of core photosystem II genes (*psbA* and *psbD*) in *Synechococcus*, whereas this phenomenon was less apparent in *Prochlorococcus* [16]. A phylogenetic analysis of *psbA* genes indicates that they were also prevalently encoded in freshwater cyanophage genomes [17], showing that both freshwater and marine cyanophages carry the necessary gene for oxygenic phototrophy. Besides the interactions between cyanobacteria and cyanophages, whole genome sequencing and metabolic reconstruction of a nitrogen-fixing marine cyanobacterium, UCYN-A, revealed a reduced genome with incomplete gene suites for the tricarboxylic acid (TCA) cycle, photosystem II, and biosynthesis of several amino acids. Nevertheless, it still has a sufficient electron transport capacity to harvest sunlight for energy generation, suggesting that UCYN-A is a photoheterotroph that is either closely associated or symbiotic with unknown partners as a result of adaptation to the oligotrophic marine environment [18]. Later in 2011, two enzymes (a novel 2-oxoglutarate decarboxylase and succinic semialdehyde dehydrogenase) that convert 2-oxoglutarate to succinate in tandem were identified in the cyanobacterium *Synechococcus* sp. PCC 7002 [19], demonstrating an alternative route completing the TCA cycle in cyanobacteria.

Studying the genomic information of environmental microbes provides DNA-based evidence of adaptive strategies for different niches. For instance, the genome sequence of *Prochlorococcus marinus* contains a low percentage of signal transduction genes, which reflects a reduced need for autotrophs in the oligotrophic ocean to be able to sense and respond to extracellular compounds [20]. Five genomic islands were identified in two *Prochlorococcus* type strains, MED4 and MIT9312 (97.9% 16S rRNA identity), using comparative genomics. These genomic islands have low coverage with the Sargasso Sea metagenome [21], and thus are presumably hotspots for genetic recombination in oceanic *Prochlorococcus* that are predisposed to carry ecologically important genes for niche differentiation [22]. The marine SAR11 *Pelagibacter ubique* possesses an unexpectedly streamlined genome by having only a few redundant DNA sequences and short intergenic lengths. Abundant ATP-binding cassette (ABC) transporters for nutrient uptake in the genome enable an adaptive strategy for oligotrophic conditions by exploiting the high substrate affinities of ABC transporters to reduce the costs of ATP hydrolysis [23]. *Silicibacter pomeroyi* (a marine *Roseobacter*) has operons for inorganic carbon monoxide and sulphide oxidation, suggesting that a lithoheterotrophic strategy is used to supplement their heterotrophy. Encoded motility genes indicate the physiological potential to find high-nutrient niches associated with particles and plankton, which is different from marine oligotrophs that subsist on dilute organic substrates [24]. Woyke *et al.* [25] applied single-cell sequencing to define the genomes of two marine flavobacteria. They used flow cytometry sorting and subsequent multiple displacement amplification to specifically amplify genomic DNA prior to whole-genome shotgun sequencing and assembly. Fragment recruitment analysis using metagenomes collected from the Global Ocean Sampling (GOS) expedition [26,27], the largest marine metagenomic library to date, revealed their numerical significance in the ocean. In contrast to their cultured relatives, the two marine flavobacteria have smaller genomes carrying fewer non-coding nucleotides and paralogous genes, suggesting genome streamlining for adaptation to narrow niches. Both genomes lacked recognizable genes for inorganic nitrogen and sulfur assimilation, suggesting a lifestyle of utilizing organic compounds as a potential energy-saving strategy for growth. For the dominant marine *Prochlorococcus*, two uncharacterized genotypes were identified through fragment recruitment using GOS metagenomic reads, and consistently dominate the surface waters of the Eastern Equatorial Pacific upwelling and tropical Indian Ocean. Analysis on the assembled genome fragments found that these two *Prochlorococcus* ecotypes lost several iron-containing proteins, indicating a genetic adaptation to the iron-depleted environments [28].

The deep sea is another unique environment in terms of low temperature and high pressure. Similar to the ocean surface, several bacteria living in the deep sea have specialized genomic features believed to be tightly associated with their adaptation to extreme conditions. The deep-sea bacterium, *Photobacterium profundum*, is a good example. High-pressure conditions not only help to optimize the swimming and swarming flagellar systems of *P. profundum* [29], but also up-regulate the expression of chitin and cellulose degradation genes [30]. A genomics analysis of the Arctic sediment bacterium, *Colwellia psychrerythraea*, found it has the ability to produce polyhydroxyalkanoates and polyamides, which is a psychrophilic lifestyle relating to the limitation of carbon and nitrogen uptake caused by cold temperatures [31]. Because cold temperatures increase oxygen levels in seawater, a remarkable strategy for avoiding reactive oxygen species generation has developed in the Antarctic psychrophile bacterium, *Pseudoalteromonas haloplanktis*, which is an alternative to the more typical molybdopterin-dependent metabolism in the genome. The higher percentage of cell envelope genes in the genome may also reflect a specialized adaptation that copes with cold temperatures and maintains normal membrane functionality [32].

## 3. Microbial Plasmid Metagenomics

While plasmid-born genes may be somewhat transient over an evolutionary time frame, they are ecologically informative and can reflect the selection pressures of the organism’s environment [33]. Studies looking into plasmid metagenomes have discovered specialized gene functions correlating with the ecology of their particular niches. An analysis of cultured marine *Roseobacter* genomes revealed that strain-specific genes concentrated on plasmids, and most of those genes were best matched to various taxa, including *Deltaproteobacteria*, *Betaproteobacteria*, *Cyanobacteria*, and *Actinobacteria* [34]. Microbial plasmids from a wastewater treatment plant were collected to assess their phylogenetic and functional diversity. By selecting for resistance to major classes of antibiotics, a cultivation-based collection of plasmid metagenomes demonstrated highly abundant reads of *Aeromonas* bacteria. In addition to classic genes for plasmid replication, stability, mobility, and transposition, the enrichment of antibiotic resistance genes indicated the reduced susceptibility of wastewater bacteria to antimicrobial drugs [35]. Similarly, the sludge microbiome also exhibited antibiotic resistance genes on their plasmids. Furthermore, the high frequency of mobile genetic elements like integrons and transposons underlined the prevalence of horizontal gene transfer in the sludge ecosystem [36]. Comparing plasmid metagenomes of two activated sludge systems receiving wastewaters from different sources, both were dominated by functions for DNA replication and transposition and harbored resistance genes against various antibiotics and heavy metals. However, differential metabolic preferences (*i.e.*, defense factors in household sludge, carbohydrate metabolism in industrial sludge) suggest a functional composition of plasmid genes associated with indigenous conditions [37]. Lastly, bovine rumen plasmid metagenomes have a highly mosaic nature in terms of their phylogenetic identity and contain abundant genes correlated with rumen niche ecology, such as sugar-utilizing enzymes, corresponding to the carbohydrate-rich rumen environment. Phylogenetic and functional diversities of plasmid genes are both believed to give advantages to their host and suggest that plasmids play a role in microbial adaptation to the environment [38].

## 4. Metabolism and Element Cycling Discovered in Metagenomics Studies

The study of metagenomes has extended our understanding of microbial ecology in various environments, adding a wealth of new information especially to microbial metabolism and its role in global geochemical cycling.

One of the key milestones is the discovery of proteorhodopsin (*i.e.*, bacterial rhodopsin) in a bacteria artificial chromosome library screen for environmental 16S rRNA sequences [39]. Proteorhodopsin is functionally a light-driven proton pump that fuels cellular processes by generating a proton motive force. The detection of proteorhodopsin’s widespread distribution in marine environments is a surprise [21], and not only demonstrates the ecological significance of this new phototrophic strategy in marine ecosystems but also explains how bacteria can be so abundant in the nutrient-limited open ocean. Subsequent investigation of proteorhodopsin indicated that a single amino residue at position 105 accounts for its spectral tuning, at which position leucine and glutamine lead to light absorption maxima at 525 nm (green light) and 490 nm (blue light) wavelengths, respectively. Proteorhodopsin variants also stratify with depth [40]. Another key discovery is the detection of ammonium monooxygenase in an archaeal genome fragment recovered from the Sargasso Sea [21,41]. This finding suggests that marine archaea might be capable of nitrification, a process previously thought to be carried out only by bacteria. In 2005, the first isolate of a marine ammonia-oxidizing archaeon, belonging to the *Crenarchaeota*, was reported [42]. A supplementary analysis of the fosmid library of marine crenarchaeon *Cenarchaeum symbiosum* successfully reconstructed the carbon and energy metabolism pathways in their genomes. Because the crenarchaeota are abundant microbes in marine ecosystems, their carbon assimilation and nitrification genes indicate important roles for these organisms in marine geochemical cycling [43].

As being one of the essential nutrients for living organisms, phosphorus (P) related pathways have also been extensively studied through metagenomics in microbes and rendered several novel insights into the microbial P-metabolisms in the environment. For example, the traditional alkaline phosphatase, PhoA, occurs much less often in the GOS metagenome than the novel PhoX phosphatase, revealing a different enzyme for marine bacterial phosphate acquisition than in cultured species [44]. A study of the subcellular localization of bacterial alkaline phosphatases (PhoA, PhoX, and PhoD) using the GOS metagenomes found that most are located in the cytoplasm. Given the prevalence of organophosphate (e.g., glycerol phosphate) uptake genes in marine bacterial genome, the cytoplasmic hydrolysis of inward-transported organophosphate would be a widespread strategy for phosphorus acquisition in sea-surface bacteria [45]. An interesting association between genomic variation and ambient phosphate concentration occurs in marine prochlorococcus. They have more genes for phosphate uptake, regulation, and utilization at sites having <0.1 μM phosphate concentrations, while most of these genes are absent in oceanic regions having >0.1 μM phosphate [46]. In the ocean, phosphonates—a group of organic phosphorus compounds containing a stable carbon-phosphorus bond—are also important phosphorus sources, although they are difficult for microbes to use. The ubiquitous distribution and active expression of phosphonoacetate hydrolase (*phnA*) in microbes along the western English Channel coast provide witness to the microbial activity of phosphonate utilization [47]. Several large-scale studies support the ecological importance of phosphonate utilization as an alternative route for phosphorus acquisition in marine bacteria by their detection of the widespread distribution of phosphonate biosynthetic and catabolic genes in the ocean [48–50]. By investigating the GOS metagenomes, *Prochlorococcus* is suggested to significantly contribute to phosphonate utilization in global surface ocean waters [51].

Dimethylsulfoniopropionate (DMSP) is a critical compound in the marine sulfur cycle. Related studies on DMSP degradation discovered two prevailing pathways in the GOS metagenomes, one being the demethylation of DMSP through DMSP demethylase (*dmdA*) [52] and the other being the cleavage of DMSP to form dimethylsulfide, the climate-changing gas, using DMSP lyase (*dddP*) [53].

## 5. Investigation of Microbial Communities Using Comparative Metagenomics

Various environmental niches have been investigated with metagenomics to determine resident community structure and metabolic potential. With the accumulated number of available metagenomes, comparative analysis has become more feasible and now increased our exploration of metagenomics at an unprecedented scale.

Enabled by the WGS approach with high-throughput sequencing, two landmark papers in metagenomics were published in 2004 describing the microbial community of the Sargasso Sea and acid mine drainage. The Sargasso Sea’s unprecedented diversity of bacteria and functional genes was reported. Over 1800 bacterial species were estimated to occur in 170–200 L of seawater, and 148 novel phylotypes and 1.2 million previously unknown genes were discovered in the Sargasso Sea surface [21]. Contrasted with the highly diverse Sargasso Sea community, acid mine drainage shows a low-complexity community dominated by a small number of species, *Leptospirillum* group II bacteria representing the most abundant members. Metabolic reconstruction derived from two nearly completed genomes of a *Leptospirillum* group II bacterium and *Ferroplasma* type II archaeon not only revealed their genomic features for adapting to the extreme environment, but also demonstrated the feasibility of genome reconstruction directly from a natural sample for the first time [54].

Habitat-specific functional fingerprints were revealed from comparisons among three disparate metagenomes taken from agricultural soil, the sea surface, and deep-sea whale carcasses. For example, proteorhodopsin, organic osmolyte transporter, and sodium export genes are abundant in the sea surface metagenome, while active potassium channeling is enriched in the soil community [55]. Foerstner, *et al.* [56] analyzed the guanine and cytosine contents (GC content) of metagenomes from four different environments and concluded that the metagenomic GC content was less relevant to the phylogenetic composition of bacterial species but seems to be actively shaped by the environment. In 2006, the first ocean interior metagenomes were reported from the Hawaii Ocean Time-series Station ALOHA. The vertical sampling design (10–4000 m) coupled with a large-scale sequencing effort (over 4500 fosmid clones) resolved some of the depth-associated functions in the ocean. Higher percentages of photosynthesis, proteorhodopsin, chemotaxis, and DNA photorepair genes were found at shallow depths, while genes for protein degradation, the degradation of small organic molecules, and polysaccharide and antibiotic production were highly represented in the deep ocean. The unexpected detection of cyanophage genes at euphotic depths presumably originated from replicating phages inside hosts, suggesting the fundamental roles of cyanophages in regulating primary production in the surface ocean [57].

Discriminatory metabolic profiles of different environments were discovered in a large-scale comparison involving 45 microbial and 42 viral metagenomes. The functional diversities of nine biomes were comparably high, but distinct metabolic preferences suggested that enriched functions are potentially predictive of environmental conditions. Viral metagenomes were described as gene repositories that support their microbial hosts’ functional versatility as they were found to have extensive numbers of microbial metabolism types [58]. In addition to metabolic profiles, a computational analysis of oligonucleotide frequency was also applied to characterize metagenomes. Among di-, tri-, and tetra-nucleotide frequencies, dinucleotide frequency achieved the highest discriminative power by explaining 80% of the variance among compared biomes, providing an alternative sequence-based metagenomic signature that can help in discriminating among environments [59].

Genomics comparison between copiotrophic (high-nutrient preferred) *Photobacterium angustum* S14 and oligotrophic (low-nutrient preferred) *Sphingopyxis alaskensis* RB2256 identified different functional preferences. Copiotrophs have more genes involved in motility, defense, transcription, and signal transduction, showing the genetic potential to be sensitive to environmental stimuli as an adaptive strategy to fluctuating nutrient supplies. However, oligotrophs have numerous genes for detoxifying various transported lipids and secondary metabolites. The prevalence of oligotrophic signature genes in the GOS pelagic and coastal metagenomes indicates the numerical dominance of free-living oligotrophs in the ocean surface [60]. Large-scale sequencing and analysis of 137 marine picoplanktonic prokaryotes (0.1–3.0 μm in size) around the globe identified two microbial groups in terms of distribution and growth strategies. One group was composed of a few cosmopolitan lineages that are nearly always abundant in the picoplankton and demonstrate a physiological preference for slow growth. The other group includes various bacterial taxa that are rarely abundant and seem capable of adjusting their growth strategy according to ambient nutrient concentrations (*i.e.*, rapid growth in high-nutrient conditions and slow growth in low-nutrient environments) [61]. Another large-scale single-cell genomics survey documented that, similar to the marine *Prochlorococcus*, *Synechococcus*, and *Pelagibacter*, strong genome streamlining and oligotrophy prevailed in other cosmopolitan planktonic bacteria in global ocean surfaces. Furthermore, the sequenced single-cell genomes recruit various amounts of metagenomic reads from global oceans and demonstrate a distribution pattern correlated with temperature and latitude. Although high 16S rRNA identities were found across different climatic zones, moderate average nucleotide similarities suggest that distributions of phylogenetically-similar lineages are not driven by dispersal limitations but more likely by environmental selection [62].

Functional metagenomics studies of oceanic regions other than the ocean surface are relatively less available. The genomic properties revealed from microbes inhabiting different oceanic regions correlate with specialized conditions of environmental niches. For example, the Lost City Hydrothermal Field is an extreme hydrothermal vent ecosystem awash in warm (40–90 °C) and highly alkaline (pH 9–10) fluids in which biofilms show low archaeal diversity in that the archaea of *Methanosaricinales* account for over 80% of the community. The high frequency of transposases on extragenomic elements might imply that rampant horizontal gene transfers in the local community are the major adaptive approach for extreme conditions [63]. The metagenome recovered from −4000 m in the Pacific Ocean also had significant enrichment in transposases and prophage sequences [64], corresponding to their discovery in hydrothermal vent biofilms. Although hydrothermal vents and bathypelagic depths are distinct from each other in terms of hydrological conditions, horizontal gene transfer seemingly appears as a conserved mechanism for microbial adaptation in contrast to ocean surface communities. The marine oxygen minimum zone (OMZ) is also a special pelagic region featuring with depleted dissolved oxygen (<20 μM). A metagenomics study investigating uncultured SUP05 gammaproteobacteria in the OMZ of Saanich Inlet, British Columbia, revealed a versatile gene repertoire mediating autotrophic carbon assimilation, sulfur oxidation, and nitrate respiration, shedding light on the metabolic insights of OMZ-associated microbiota [65]. A recent report investigating particle-associated metagenomes in the OMZ off the coast of Iquique, Chile, reported that genes for nitrate reduction were detected more in the larger-sized fraction of the community (>1.6 μm), while those for sulfur metabolism had a distribution less relevant to particle size [66].

The complete set of mRNA sequences (or transcripts) from a given community is called a metatranscriptome, and studying metatranscriptomes (*i.e.*, metatranscriptomics) represents a distinct approach from metagenomics to directly monitor potential biological activities rather than the static genomic contents of a community. Supplementing metatranscriptomics data to metagenomes also helps in the quantitative identification of active phylotypes and associated metabolic functions. A metatranscriptomic study examining the community in the oligotrophic Pacific Ocean surface found that cyanobacterial and alphaproteobacterial transcripts were predominant and most transcripts encoded genes of photosynthesis, carbon fixation, and nitrogen acquisition. Aligning metagenomic reads and metatranscriptomic transcripts with the reference *Prochlorococcus* genome revealed that the five previously-discussed genomic islands [22] were covered by low numbers of DNA reads but high numbers of cDNA transcripts, highlighting the importance of these genomic islands for sea-surface adaptation [67]. Another metatranscriptomics study contrasting day and night gene expression profiles in eight oceanic regions discovered that 74% of expressed functions were shared by their microbial communities and the remaining variability could be explained by assemblage differences. For instance, the differential abundance of transcripts for photosynthesis might correspond to the site-to-site variability among photosynthetic microbial populations [68].

## 6. Decoupling of Community and Functional Diversity

A microbial community has two main characteristics: community composition (who they are) and embedded functional traits (what they can do). The association between these two has been discussed in various reports. In a manipulative experiment, community composition was proposed to be important in determining ecosystem functioning [69]. Following that, several environmental genomics surveys also explored how a community composition correlates with its functional traits at different temporal and spatial scales. Communities collected from seven oceanic sites along the meridional transect from North Pacific to South Pacific subtropical gyres showed different phylogenetic compositions, while metabolic potentials encoded in the metagenomes were very similar, suggesting that a core suite of functional genes may be present in the genomes of dominant microbes [70]. A community survey tackling bacterial metagenomes associated with the green macroalga *Ulva australis* discovered that the communities were highly diverse in phylogenetic composition, but their functional traits were pretty similar from one sample to another. Therefore, the authors concluded that the main characteristic describing a bacterial assemblage is its group of functional genes rather than the species that composed it [71]. A long-term metagenomics study comparing microbial community composition and functional profiles also found that the variation among observed bacterial genera composition was three-fold higher than that in Clusters of Orthologous Groups family profiles, indicating the unsynchronized nature of community and functional dynamics [72]. Experimental verification from soil and sediment samples taken along a stream corridor similarly indicated that seasonal variations in bacterial community composition (revealed by denaturing gradient gel electrophoresis) were unrelated to the ecosystem functions (defined by enzyme activity assay) of carbon, nitrogen, and phosphorus acquisition [73]. Therefore, the decoupling of community structure and functional diversity is a prominent feature of microbial communities and is correlated to functional redundancy in the genomes of phylogenetically different microbes [74].

## 7. Conclusions

The study of either a single genome or an entire metagenome can reveal specialized gene suites correlating with different environments such as the ocean surface and cold deep waters. Discoveries in metabolic pathways and enzymes demonstrate the power of metagenomics, which has greatly improved our knowledge of microbial ecology in the natural environment. Several genomics patterns have repeatedly been found throughout the globe and are summarized herein. To begin with, genome reduction in free-living bacteria in ocean surfaces is a major adaptive strategy for the oligotrophic open-ocean environment, which accounts for the dominance of oligotrophic lineages in the epipelagic layer, while horizontal gene transfer is a typical genomic activity in the deep-sea community. Aside from that, environmental niches tightly associate with the metabolic capability (or functional profiles) encoded in indigenous metagenomes, representing a genomic signature—like nucleotide frequency—that can be predicted for different types of ecosystems. Likewise, at the single genome level, a specific trophic strategy can be implied by a particular functional gene composition. As being one part of the microbial genome, genes on plasmids can also be controlled by an environmental niche. Lastly, decoupling between community structure and functional diversity could be more widespread than previously thought, which warrants further investigation.
